# Gender-specific effects of smoking and alcohol consumption on cardiometabolic diseases and multimorbidity: A cross-sectional study

**DOI:** 10.18332/tid/208109

**Published:** 2025-09-19

**Authors:** Pei Sun, Jie Gao, Xiao Liang, Xin Zhang, Xiao Zhang, Xiaopeng Yan, Chunping Ni

**Affiliations:** 1 Department of Basic Nursing, School of Nursing, Air Force Medical University, Xi'an, China

**Keywords:** lifestyle, cardiometabolic diseases, multimorbidity, gender specific

## Abstract

**INTRODUCTION:**

Gender-specific variations in hormonal profiles, adipose tissue distribution, and metabolic pathways may differentially modulate the health impacts of smoking and alcohol use. Current population-based studies on the impact of smoking and alcohol consumption on cardiometabolic diseases (CMD) and multimorbidity (CMM) often lack gender-stratified analyses, thereby limiting the evidence base for gender-tailored preventive strategies.

**METHODS:**

This population-based cross-sectional study used data from the 2020 China Health and Retirement Longitudinal Study (CHARLS), and a total of 11447 participants were included in the analysis. Multinomial logistic regression was conducted to assess behavioral risk factors, with interaction terms evaluating effect modification by gender.

**RESULTS:**

The prevalence was 16.67% for CMD and 5.66% for CMM. Participants who smoked were more likely to report CMM than those who did not smoke (OR=2.70, p<0.05). Smoking was significantly associated with the prevalence of CMD in females (AOR=1.34, p<0.05), but not in males (p=0.556). Moreover, female smokers were more likely to report CMM compared to male smokers (AOR_females_=3.53, AOR_males_=2.02, p<0.05). No significant associations were found between alcohol consumption and the prevalence of CMD or CMM, nor were any gender-specific differences observed.

**CONCLUSIONS:**

Smoking may have a potential gender-specific effect on the risk of CMD and CMM, with female smokers exhibiting a higher prevalence of CMM than males. This highlights the need to integrate gender considerations into chronic disease prevention frameworks.

## INTRODUCTION

Cardiometabolic diseases (CMD), encompassing hypertension, dyslipidemia, diabetes, and cardiovascular diseases, have emerged as a growing public health challenge among middle-aged and elderly populations globally^1-3^. Individuals with two or more concurrent CMDs are clinically defined as having cardiometabolic multimorbidity (CMM). A cohort study involving 689000 participants indicated that the risk of mortality escalates exponentially with the number of CMDs present^4^. The etiology of CMD and CMM involves multiple factors, including lifestyle behaviors and genetic predisposition^5-7^, among which modifiable behaviors like smoking and alcohol consumption represent key preventable risk factors^8-10^.

Gender-specific variations in hormonal profiles, adipose tissue distribution, and metabolic pathways may differentially modulate the health impacts of smoking and alcohol use^11-13^. For example, women exhibit heightened alcohol sensitivity due to reduced gastric alcohol dehydrogenase activity and diminished firstpass metabolism^14^. And compared to males, females exhibit faster nicotine metabolism rates due to higher activity of CYP2A6 – the primary enzyme responsible for nicotine metabolism – mediated by female sex hormones^15^. Despite these mechanistic insights, current population-based investigations frequently neglect gender-stratified analyses, which limits evidence for gender-tailored preventive strategies.

Accordingly, this study employs data from CHARLS to investigate gender-specific associations between smoking/alcohol consumption patterns and both incident cardiometabolic diseases and multimorbidity among middle-aged and older adults. Our findings aim to inform precision lifestyle interventions that target this high-risk demographic.

## METHODS

### Data sources

This cross-sectional study utilized data from the 2020 wave of CHARLS which is a nationally representative longitudinal survey focusing on the socioeconomic and health status of Chinese adults aged ≥45 years. The 2020 wave of CHARLS was conducted between April and September 2020. The survey employed a stratified multistage probability sampling design, covering 150 county-level units across 28 provinces in China, thereby demonstrating strong representativeness of the middle-aged and elderly population. The CHARLS was approved by the Biomedical Ethics Review Committee of Peking University. All participants provided written informed consent. The ethical approval number of CHARLS is IRB00001052-11015.

The initial dataset included 19395 participants. After sequentially excluding those who were: 1) missing key covariates (gender, age, residence) (n=55); 2) aged <45 years (n=237); 3) lacking information on CMD (n=7947); and 4) lacking complete smoking/alcohol data (n=159), the final analytical sample yielded 11447 eligible individuals.

### Variables

The response variable in this study was the presence or absence of any CMDs. Similar to the previous study^16^, CMDs were defined as hypertension, dyslipidemia, diabetes, heart disease, and stroke for this study. CMD was assessed by the following questions: ‘Have you been diagnosed with the conditions listed below by a doctor: 1) hypertension; 2) dyslipidemia; 3) diabetes or high blood glucose; 4) heart attack, coronary heart disease, angina, congestive heart failure, or other heart problems; and 5) stroke’. The CMM represented the coexistence of a minimum of two CMDs^4^.

The exposure variables are smoking and alcohol consumption behaviors. Smoking behavior was assessed by the question: ‘Do you currently smoke? (including cigarettes, hand-rolled tobacco, pipe smoking, or chewing tobacco)’. Responses of ‘Yes’ were defined as smoking. Alcohol consumption behavior was assessed by the question: ‘In the past year, have you consumed alcohol, including beer, wine, rice wine, yellow rice wine, Chinese liquor, medicinal liquor, etc.?’. Responses of ‘Yes’ were defined as alcohol consumption.

Based on prior research^17^, the control variables in this study included age, gender, marital status, residence, and other chronic diseases. Chronic diseases encompassed malignant tumors, pulmonary diseases, liver diseases, kidney diseases, memory-related disorders, Parkinson’s disease, etc. Respondents who reported having one or more of the aforementioned conditions were defined as having other chronic diseases.

### Data analysis

Microsoft Excel 2021 and SPSS 26.0 were employed for the extraction, transformation, coding, and analyzing of variables. Categorical variables are presented as frequencies and percentages. Continuous variables were assessed for normality using the Kolmogorov-Smirnov test and are reported as mean ± standard deviation (SD) if they followed a normal distribution; otherwise, they were expressed as median with interquartile range (IQR). The chisquared test was used to compare participants’ general characteristics according to gender.

Collinearity diagnostics showed no multicollinearity (tolerance>0.1, VIF <10 across all variables). The parallel line test for ordinal logistic regression was statistically significant (p<0.05), indicating a violation of the proportional odds assumption. Therefore, multinomial logistic regression was employed to investigate the associations of smoking, alcohol consumption, and other covariates with the prevalence of CMD and CMM, with non-CMD individuals as the reference group. The interaction terms gender × smoking and gender × alcohol were also included as independent variables in the multinomial logistic regression model for analysis. Maximum Likelihood Estimation (MLE) was subsequently applied for parameter estimation, and Likelihood Ratio Tests (LRT) were conducted to assess potential interaction effects between smoking/alcohol consumption behaviors and gender. All tests were two-tailed, and statistical significance was set at p<0.05.

## RESULTS

### The characteristics of participants

**Figure 1:**
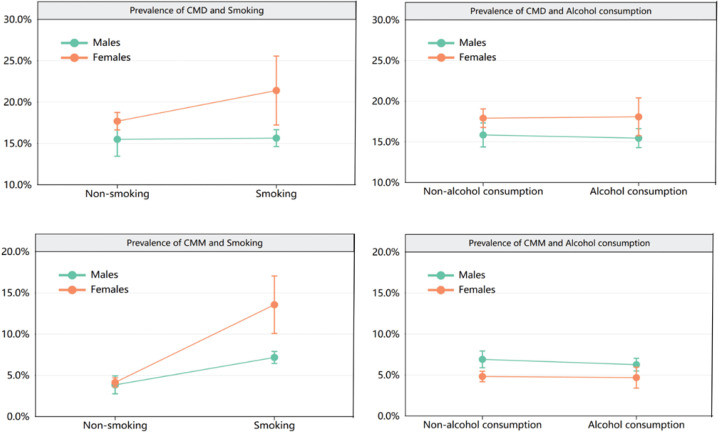
Prevalence with 95% CIs of CMD and CMM by gender and smoking/alcohol consumption behaviors among adults aged ≥45 years, CHARLS, 2020 (N=11447)

The study involved 11447 individuals with a median age of 59 years (IQR: 53–66). Among the participants, there were 6053 males (52.88%) and 5394 females (47.12%). The prevalence of CMD was 16.67%, and that of CMM was 5.66%. The characteristics of participants according to gender are presented in Table 1. Figure 1 shows the prevalence of CMD with 95% CIs by gender and smoking/alcohol consumption behaviors.

**Table 1:** Characteristics of participants according to the gender, CHARLS, 2020 (N=11447)

*Characteristics*	*Males* *n (%)*	*Females* *n (%)*	*p*
**Age** (years)			<0.001
45–64	3942 (65.12)	3930 (72.86)	
65–79	1987 (32.83)	1385 (25.68)	
≥80	124 (2.05)	79 (1.46)	
**Residence**			<0.001
City	1567 (25.89)	1734 (32.15)	
Town	774 (12.79)	727 (13.48)	
Rural	3712 (61.32)	2933 (54.38)	
**Marital status**			<0.001
Married	5554 (91.76)	4584 (84.98)	
Single/divorced/widowed	499 (8.24)	810 (15.02)	
**Smoking**			<0.001
Yes	4854 (80.19)	368 (6.82)	
No	1199 (19.81)	5026 (93.18)	
**Alcohol consumption**			<0.001
Yes	3706 (61.23)	1050 (19.47)	
No	2347 (38.77)	4344 (80.53)	
**Number of prevalences of CMD**			<0.001
0	4718 (77.94)	4172 (77.35)	
1 (CMD)	943 (15.58)	966 (17.91)	
≥2 (CMM)	392 (6.48)	256 (4.75)	
**Having other chronic diseases**			0.004
Yes	4718 (77.94)	4172 (77.35)	
No	943 (15.58)	966 (17.91)	

### Factors associated with CMD and CMM

Table 2 presents the associations of smoking, alcohol consumption behaviors, and demographic variables with the prevalence of CMD and CMM. The results showed that age, gender, and the presence of other chronic conditions were significantly associated with the prevalence of CMD (all p<0.05). Residence, marital status, smoking status, and presence of other chronic conditions showed significant associations with the prevalence of CMM (all p<0.05).

**Table 2:** Multinomial logistic regression results on factors associated with CMD and CMM, CHARLS, 2020 (N=11447)

Variables	CMD		CMM	
	OR (95% CI)	p	OR (95% CI)	p
**Age** (years)				
45–64 ^®^	1		1	
65–79	1.32 (1.19–1.48)	<0.001**	1.14 (0.96–1.37)	0.130
≥80	0.92 (0.62–1.37)	0.695	0.63 (0.32–1.27)	0.197
**Residence**				
Rural ^®^	1		1	
City	1.05 (0.94–1.18)	0.328	1.37 (1.14–1.63)	0.001*
Town	1.06 (0.91–1.24)	0.402	0.98 (0.76–1.27)	0.927
**Marital status**				
Single/divorced/widowed ^®^	1		1	
Married	0.86 (0.74–1.01)	0.075	0.75 (0.59–0.95)	0.022*
**Gender**				
Male ^®^	1		1	
Female	1.28 (1.09–1.49)	0.002*	1.42 (1.09–1.85)	0.008*
**Smoking**				
No ^®^	1		1	
Yes	1.13 (0.98–1.32)	0.091	2.70 (2.10–3.48)	<0.001**
**Alcohol consumption**				
No ^®^	1		1	
Yes	0.99 (0.76–1.27)	0.950	0.87 (0.73–1.04)	0.142
**Having other chronic diseases**				
No ^®^	1		1	
Yes	1.85 (1.65–2.07)	<0.001**	2.82 (2.39–3.34)	<0.001**

* p<0.05.

** p<0.01.

^®^ Reference categories.

### Interaction effect analysis

After adjusting for potential confounders, including age, residence, marital status, and the presence of other chronic diseases, smoking and the gender × smoking interaction term were significantly associated with the prevalence of CMD, as shown in Table 3. The likelihood ratio test for model effects revealed that the gender × alcohol interaction term was not statistically significant (χ^2^=0.034; p=0.983), whereas the gender × smoking interaction term was statistically significant (χ^2^=8.005; p=0.027).

**Table 3:** Interaction effects of gender with smoking and alcohol consumption on CMD and CMM, CHARLS, 2020 (N=11447)^a^

Variables	CMD		CMM	
	AOR (95% CI)	p	AOR (95% CI)	p
**Gender**				
Male ^®^	1		1	
Female	1.18 (0.97–1.43)	0.093	1.05 (0.73–1.49)	0.790
**Smoking**				
No ^®^	1		1	
Yes	1.05 (0.88–1.26)	0.130	2.03 (1.47–2.79)	<0.001**
**Alcohol consumption**				
No ^®^	1		1	
Yes	0.98 (0.85–1.14)	0.941	0.87 (0.70–1.07)	0.200
**Interaction terms**				
Gender × smoking	1.28 (0.92–1.76)	0.130	1.82 (1.13–2.91)	0.012*
Gender × alcohol	1.02 (0.81–1.28)	0.941	1.01 (0.68–1.49)	0.940

a Model was adjusted for age group, residence, marital status, and other chronic diseases. AOR: adjusted odds ratio.

^®^ Reference categories.

* p<0.05.

** p<0.01.

Subgroup analysis was conducted according to gender. The results showed that, in males, smoking was associated with a higher prevalence of CMM (AOR=2.02; 95% CI: 1.46–2.78; p<0.001). In females, smoking was associated with a higher prevalence of CMD (AOR=1.34; 95% CI: 1.02–1.75; p=0.034) as well as CMM (AOR=3.53; 95% CI: 2.50–4.99; p<0.001). The results are presented in Table 4.

**Table 4:** Associations of smoking/alcohol consumption with CMD and CMM by gender, CHARLS, 2020 (N=11447)^a^

Gender	Variables		CMD		CMM	
			AOR (95% CI)	p	AOR (95% CI)	p
**Male**	Smoking	No ^®^	1		1	
		Yes	1.05 (0.86–1.25)	0.556	2.02 (1.46–2.78)	<0.001**
	Alcohol consumption	No ^®^	1		1	
		Yes	1.00 (0.86–1.11)	0.989	0.88 (0.71–1.09)	0.272
**Female**	Smoking	No ^®^	1		1	
		Yes	1.34 (1.02–1.75)	0.034*	3.53 (2.50–4.99)	<0.001**
	Alcohol consumption	No ^®^	1		1	
		Yes	1.03 (0.86–1.23)	0.700	0.92 (0.66–1.29)	0.660

a Model was adjusted for age group, residence, marital status, and other chronic diseases. AOR: adjusted odds ratio.

* p<0.05.

** p<0.01.

^®^ Reference categories.

## DISCUSSION

Based on the 2020 data from the China Health and Retirement Longitudinal Study (CHARLS), we explored the impact of smoking and alcohol consumption behaviors on the prevalence of cardiovascular metabolic diseases and their comorbidities among middle-aged and elderly Chinese and analyzed gender differences in related health risks. Our results showed that smoking was significantly associated with a higher prevalence of CMM, and there was an interaction between gender and smoking behavior; specifically, female smokers had a significantly higher prevalence of CMM compared to male smokers. In contrast, alcohol consumption was not significantly associated with the prevalence of CMM, and no gender-specific effects were observed.

Our study results showed that smoking demonstrated a significant association with a higher prevalence of CMD and CMM in females than males. Li et al.^18^ found that smoking is associated with the prevalence of heart disease, and its effect is more pronounced in females. Yang et al.^19^ found that female smokers with myocardial infarction have worse prognoses and higher mortality rates compared to male smokers. These findings are consistent with the results of the present study. The disparities in hormonally mediated mechanisms and nicotine metabolism may represent potential causes for the observed results. A study found that, due to the influence of estrogen, smoking causes a more significant increase in low-density lipoprotein levels in females than in males, predisposing females to dyslipidemia^20^. Furthermore, other researchers have found that smoking has a greater impact on increasing serum uric acid levels in female patients^21^, which may contribute to atherosclerotic plaque formation and vascular intimal injury^22^. Gender differences also exist in tobacco metabolism, as females extract relatively higher levels of carcinogens and other toxic compounds from the same number of cigarettes compared to males^15,23^. Although smoking prevalence is substantially higher among males than females globally, it is noteworthy that both our findings and related studies indicate that smoking inflicts greater physiological and psychological harm on females^24^. Furthermore, research suggests gender differences exist in tobacco’s effects on the brain reward system, with females facing greater challenges during smoking cessation attempts^25^. Currently, tobacco control policies in China and other countries are increasingly implemented^26,27^. However, females remain an underrecognized factor in both policy formulation and execution^28^. This highlights the need to strengthen targeted health promotion efforts for middle-aged and older females within current tobacco control strategies.

As indicated by research findings such as those from Tonnesen et al.^29^, the occurrence of new cardiovascular events is a significant predictor of smoking cessation. Our analysis may exist a reverse causation: individuals who develop CMD due to smoking subsequently quit smoking. This diseasedriven smoking cessation behavior may dilute the true harmful effect of smoking, thereby masking the strength of the association we observed. Consequently, although a significant association was not found, our results do not imply the absence of harm from smoking.

Alcohol consumption in this study was not significantly associated with the prevalence of developing CMD or its comorbidities among middleaged and older adults. The relationship between alcohol intake and cardiovascular metabolic outcomes remains a subject of diverse perspectives. The predominant view suggests a U-shaped or J-shaped dose-response curve between alcohol consumption and cardiovascular disease, wherein low-to-moderate alcohol intake – corresponding to the nadir of the curve – may confer a protective effect^30,31^. This phenomenon has similarly been observed in studies examining the associations between alcohol consumption and both diabetes and dyslipidemia^32,33^. Given the absence of detailed alcohol consumption data within the CHARLS database, our study could not further analyze the differential impacts of various drinking patterns on cardiovascular metabolic diseases. It is plausible that distinct drinking patterns simultaneously exert both protective and detrimental effects, and the counteraction between these opposing influences may partially explain the lack of significant associations between alcohol consumption and CMD/CMM prevalence observed herein.

### Strengths and limitations

Our study analyzed data from 11447 participants in the CHARLS database, thus providing a degree of population representativeness. However, our study has several limitations. First, the cross-sectional design of the study may limit causal inference. Second, selfreported data on smoking, alcohol consumption, and disease status may introduce recall bias. Third, there were some potential but unmeasured confounding variables in this study, such as physical activity, mental health status, or nutritional status. Finally, we did not differentiate between types or doses of tobacco and alcohol products, which may obscure important exposure-response relationships.

## CONCLUSIONS

Smoking may have a potential gender-specific effect on the risk of CMD and CMM; female smokers show a significantly higher prevalence of CMM than male smokers. Our findings recommend integrating gender considerations into chronic disease prevention and control systems, and enhancing early screening and intervention for female smokers. Alcohol consumption did not show significant gender-specific interaction effects on the prevalence of CMD or CMM. Furthermore, it remains necessary to continuously monitor the long-term health impacts of different patterns of alcohol consumption.

## Data Availability

The data used in this study are publicly available through the China Health and Retirement Longitudinal Study (CHARLS) database, which is accessible at: http://charls.charlsdata.com Access to the CHARLS dataset requires registration and approval from the CHARLS project team, and users must adhere to the terms of use set by the database.
